# Association of fruits and vegetables consumption and related-vitamins with inflammatory and oxidative stress markers in prediabetic individuals

**DOI:** 10.1186/1758-5996-6-22

**Published:** 2014-02-18

**Authors:** Luciana Dias Folchetti, Milena Monfort-Pires, Camila R de Barros, Lígia Araújo Martini, Sandra Roberta Gouvea Ferreira

**Affiliations:** 1 Departamento de Nutrição, Faculdade de Saúde Pública da Universidade de São Paulo, Av. Dr. Arnaldo, 715, São Paulo, SP CEP 01246-904, Brazil

**Keywords:** Fruits and vegetables, Vitamins, Oxidative stress, Insulin resistance, Inflammation

## Abstract

**Background:**

Dietary guidelines of 5 servings per day of fruits and vegetables (FV) offer a reasonable amount of vitamins to control organic processes, which may contribute to a favorable cardiometabolic profile. This study aimed at investigating whether the intake of the FV group as well as pro-vitamin A carotenoids and vitamins C and E were associated with circulating markers of oxidative stress, inflammation and insulin resistance in Brazilians individuals at cardiometabolic risk.

**Methods:**

This cross-sectional study included 205 individuals screened for diabetes prevention program in a healthcare center from the School of Public Health, University of São Paulo, conducted in 2008. Possible associations of consumption of FV group, as well as pro-vitamin A carotenoids and vitamins C and E, with circulating markers of oxidative stress (superoxide dismutase – SOD and oxidized LDL – oxLDL), inflammation (C reactive protein, TNF-α and adiponectin) and insulin resistance (HOMA-IR) were investigated. Pearson correlation coefficient, ANOVA and multiple linear regression were employed.

**Results:**

The sample (64.7% women) had a mean age of 54.1 ± 12.7 years and body mass index of 30.7 ± 5.7 kg/m^2^. Dietary, physical activity, anthropometric and laboratory data were obtained. Participants consumed a mean of 3.8 servings/day of FV; their FV intake was categorized into three groups: <2.5, 2.5-5.0 and >5.0 servings/day. Significant trends for lower waist circumference (103.4 ± 13.6 vs. 100.1 ± 12.2 vs. 98.2 ± 12.7 cm, p-trend <0.05) and higher adiponectin concentrations (10.4 ± 1.8 vs. 11.9 ± 1.9 vs. 13.6 ± 2.1 ng/mL, p-trend <0.05) were detected across categories. Associations between SOD concentrations (β 0.172 [0.110-0.688]) with FV consumption and between oxLDL concentrations with vitamins C (β -0.333 [−2.568 – -0.218]) and E (β -0.354 [−1.131– -0.110]) intakes, adjusted for age, gender, BMI, saturated fat intake, smoking and physical activity were found. Similar results were observed for the associations between oxLDL and FV intake, but significance disappeared adding adjustment for saturated fat, smoking and physical activity.

**Conclusion:**

Our data suggest that the intake of FV or selected vitamins may be useful for identifying the oxidative stress and inflammation involved in the genesis of cardiometabolic diseases and for motivating at-risk patients for changing dietary habits.

## Background

Dietary habits have been involved in the pathophysiology of diseases. Lifestyle interventions have confirmed that a healthy diet combined with physical activity are able to improve underlying mechanisms of metabolic and cardiovascular diseases, such as oxidative stress, inflammation and insulin resistance [1,2]. Several benefits from a fiber-rich diet including fruits & vegetables (FV) on health have been described. International recommendations of 5 servings a day of this food group are based also on their high micronutrient content, which can induce favorable cardiometabolic outcomes [3,4]. High FV consumption has been associated with decreased incidence and mortality from a variety of obesity-related diseases including type 2 diabetes and cardiovascular diseases [2,5-7], which are conditions of oxidative stress and chronic inflammation [2,4,7,8]. These associations have been attributed either as to an interaction between micro and macronutrients, or to nutrients in isolation.

Pro-vitamin A carotenoids and vitamins C and E, present in FV, are essential for proper physiological functioning. The importance of vitamin E for maintaining oxidative-antioxidant balance is widely recognized [8,9], but this must be accompanied by vitamin C in order to enhance antioxidant protection [8-10]. Pro-vitamin A carotenoids are present in brightly colored FV; such micronutrients modulate immune system and exert a protective action by reducing LDL-cholesterol oxidation via induction of antioxidant enzymes [10,11]. Although the synergism of different nutrients consumed in a daily diet could limit the ability to separate out specific effects, reported in observational studies [12], it was of our interest investigating how the intake of those particular vitamins or their main souces (FV) could be associated with circulating markers of oxidative stress and inflammation, then contributing to increase cardiometabolic risk.

The reported benefits of FV on health are in part dependent on their content of natural antioxidant as vitamins A, C, and E, since oxidative stress is an underlying mechanism for several chronic diseases [9,13,14]. Studies in animal models and humans have provided evidence about the pathways by which micronutrients in FV may induce protective cardiometabolic effects [4,7,15]. Unbalance between free radicals production and antioxidant enzymes causes damage of multiple cellular components and even death [13,16], and it would be expected that restoring redox equilibrium could attenuate the risk of diseases such as type 2 diabetes and atherosclerosis [15-17]. However, multivitamins supplementation in randomized trials have not proven to reduce cardiovascular morbidity and mortality [9,13,16]. Considering that high doses of certain vitamins may adversely affect lipid concentrations, natural intake through a healthy diet has been recommended [13,16].

Several circulating biomarkers have been used to indicate *in vivo* oxidative stress (urinary F2-isoprostanes and oxidized LDL – oxLDL) or antioxidant defense (glutathione peroxidase, superoxide dismutase – SOD or total antioxidant capacity), although none is considered the gold standard [4,8,10,15]. It is known that oxidative stress alters intracellular signaling pathways inducing insulin resistance, a process which is aggravated by oxidative stress-triggered inflammatory mechanisms [15,17]. A pro-inflammatory state has been detected by elevated C-reactive protein (CRP) concentrations. Tumor necrosis factor alpha (TNF-α) is a major cytokine that affects phosphorylation of IRS-1 into serine and GLUT4 translocation, reducing cellular glucose uptake [18]; TNF-α mediates the production of proteins either with pro- or with anti-inflammatory properties, such as adiponectin [19,20]. Disturbances in these mechanisms are not routinely evaluated in clinical practice. Considering that dietary habits influence these mechanisms, assessment of food consumption could provide indirect way to evaluate oxidative-antioxidant balance and inflammatory status.

In humans, beneficial impact of FV intake on the incidence of type 2 diabetes was reported [5,6]. However, a study conducted in Brazil suggested that high intake of fruits and juices could favor glucose metabolism disturbances [21], calling attention that analysis of different contents in food groups could result in contrasting effects. In addition, different populations may also respond distinctly due to the cardiometabolic risk level and/or genetic background.

We raised the hypothesis that dietary assessment by the FV group and/or vitamins could provide valuable information to help identify relationships between consumption and certain biological disturbances in a sample of Brazilian population, known to be highly miscegenated. This study aimed at investigating whether the intake of the FV group as well as pro-vitamin A carotenoids and vitamins C and E were associated with circulating markers of oxidative stress (oxidized LDL and superoxide dismutase), inflammation (CRP, TNF-α and adiponectin) and insulin resistance (HOMA-IR) in Brazilians individuals at cardiometabolic risk.

## Material and methods

### Study population

This study was approved by the Research Ethics Committee of the School of Public Health of the University of São Paulo (FSP-USP), Brazil. Written consent was obtained from all participants.

A total of 438 individuals of both sexes aged 21 to 79 years, assisted by the FSP-USP school health center was screened for a type 2 diabetes prevention program and invited to participate in a lifestyle intervention, registered at http://www.ensaiosclinicos.gov.br (RBR #65 N292).

Baseline data were collected between 2008 and 2009. Two-hundred thirty individuals were selected for the intervention program, after applying eligibility criteria. Inclusion criteria were adults with prediabetes (impaired glucose tolerance or impaired fasting glycemia) according to American Diabetes Association diagnostic criteria [22]. Individuals with a medical history of neurological or psychiatric disturbances, thyroid, liver, renal or infectious diseases and those taking vitamins supplements were excluded. For the purposes of the present study, the absence of dietary data, biomarkers determinations and confounding variables was also exclusion criterion; 205 individuals had all the data necessary for the current analysis.

### Data collection

A multiprofessional team (endocrinologist, nutritionist, physical educator and psychologist) was trained to fill specific questionnaires and electronic entries were double-checked. Data on sociodemographic characteristics, smoking and other habits or health issues were obtained through standardized questionnaires. Physical activity was assessed by the long version of the international physical activity questionnaire [23]. Dietary data were collected using three 24-h dietary recalls for each individual applied by trained nutritionists. The three recalls were applied on non-consecutive days, two on weekdays and one at the weekend, and means of consumptions for each nutrient were calculated. The first recall was obtained by direct interview, and the others collected by telephone. Data on food intake were recorded in household measures and converted to their respective weights and volumes (grams or milliliters), based on Brazilian food composition tables [24], and processed on the Nutrition Data System® (NDS - NCC, 2005), which contains a large database of foods and their components. For each day, total amounts of retinol, total tocopherols and ascorbic acid, present in all the food sources were obtained. Based on reports provided by the software, the FV food group was created, including fruits (apple, avocado, banana, berries, cherries/plum, citrus fruits, guava, grapes, kiwi, mango, melons, papaya, peach/apricot/nectarine, pear, passion fruit, persimmon, pineapple and plum) and fresh juices of these fruits and along with green leafy, cruciferous, carotenoid-containing and miscellaneous vegetables. Intake data were then transformed into servings according to the Food Guide for the Brazilian population.

Height was measured using a fixed stadiometer and weight was taken with individuals wearing light clothes and no shoes on a Filizola digital scale with a capacity of 200 kg and accurate to the nearest 100 g. Body mass index (BMI) was calculated as weight (kilograms) divided by height in meters squared. Waist circumference was measured at the midpoint between the bottom of the rib cage and above the top of the iliac crest during minimal respiration. Bioelectrical impedance analysis was measured using the Quantum II - BIA Analyzer (RJL Systems, Inc., Clinton Township, Michigan, USA).

### Laboratory

Fasting blood samples were obtained for plasma glucose and lipoproteins determinations, performed immediately in the local laboratory using validated commercial kits. The samples were centrifuged and aliquots stored at −80°C for further determinations of superoxide dismutase (SOD), oxidized LDL (oxLDL), inflammatory markers and hormones. SOD was determined using a commercial kit (Assay Designs, Inc, Ann Arbor, Michigan, USA) and Oxidized Low-Density Lipoprotein/β2-Glycoprotein I (oxLDL-β2GPI human) by an ELISA kit (Cayman Chemical Company, Ann Arbor, Michigan, USA. Intra-assay coefficients of variability ranged from 5.3% to 7.4% and the inter-assay coefficient of variability from 5.9% to 8.4%. CRP (reference < 0.3 mg/dL) and TNF-α were measured using immunoenzyme chemiluminescent assay (Immulite, Diagnostic Products Corporation, Los Angeles, CA, USA). Insulin was determined by immunometric assay using a quantitative chemiluminescent kit (AutoDelfia, Perkin Elmer Life Sciences Inc, Norton, OH, USA) and adiponectin was measured by enzyme-linked immunosorbent assay (Linco Research, St. Charles, Missouri, USA). Homeostasis model assessment (HOMA-IR) was used to assess insulin resistance [15]; a previously described reference cutoff for a Brazilians was considered [25].

### Statistical analysis

All statistical analyses were performed using the Statistical Package for Social Sciences (SPSS v. 17.0) software program. Distributions of HOMA-IR, CRP, TNF-α, adiponectin, SOD, oxLDL and micronutrients were skewed and thus transformed before analysis to achieve normality (logarithmic or square root transformation). Means and standard deviations were back-transformed to return to the natural scale. To increase precision of the nutrient and food intake analyses, an average of the dietary data collected on the three recalls was obtained. Subsequently, the number of servings was calculated. Only for analysis purposes, nutrients were adjusted for 1000 kcal as an attempt to minimize inter-individual variation [26].

Clinical and dietary data were given according to gender. Participants’ intakes of micronutrients were analyzed according to the Dietary Reference Intakes – DRIs [27,28]. FV intake was categorized into three groups: below 2.5, between 2.5 and 5.0, and above 5.0 servings per day; intake of micronutrients was divided into tertiles. These groups were compared using ANOVA and p for trend was calculated. Pearson’s coefficient was used to test correlations. Multiple linear regression analysis was used to test independent associations of intakes of FV or vitamins A, C and E (dependent variables) with markers of inflammation, oxidative stress and insulin resistance (independent variables). Coefficients (95% confidence interval provided) were adjusted for potential confounders, such as age, gender, BMI (model 1) saturated fat acid intake, smoking status and physical activity (model 2). A p-value < 0.05 was considered significant.

## Results

The mean age of the study sample (64.7% women) was 54.1 ± 12.7 years; 33% were Caucasians and 80% non-smokers. Clinical and dietary data according to gender are shown in Table 1. Despite similar BMI and waist circumference in both genders, women showed higher percentage of fat mass (38.4 ± 6.8 vs. 25.8 ± 6.3%, p < 0.01). Also, adiponectin (13.7 ± 1.8 vs. 8.7 ± 1.9 ng/mL, p < 0.01) and CRP concentrations (0.4 ± 0.1 vs. 0.2 ± 0.1 mg/dL, p < 0.01) were significantly greater in women than in men.

**Table 1:** Mean values (standard deviation) of clinical and dietary data of the 205 participants according to gender

	**Men**	**Women**	**P value**
**• Clinical data**			
Body mass index (kg/m^2^)	29.1 ± 4.5	31.3 ± 5.8	0.05
Waist circumference (cm)	102.7 ± 12.5	100.0 ± 13.2	0.16
Fat mass (%)	25.8 ± 6.3	38.4 ± 6.8	<0.01
Fasting plasma glucose (mg/dL)	101.2 ± 10.6	98.3 ± 11.8	0.08
Non-HDL cholesterol (mg/dL)	153.9 ± 38.9	157.6 ± 43.6	0.55
Triglycerides (mg/dL)	161.5 ± 69.4	144.3 ± 67.1	<0.01
Adiponectin (ng/mL)	8.7 ± 1.9	13.7 ± 1.8	<0.01
HOMA-IR	1.9 ± 1.9	2.0 ± 1.9	0.52
C-reactive protein (mg/dL)	0.2 ± 0.1	0.4 ± 0.1	<0.01
Tumor necrosis factor-α (pg/mL)	12.0 ± 4.7	12.5 ± 7.4	0.59
Superoxide dismutase (U/ml)	44.2 ± 1.5	42.6 ± 1.4	0.50
Oxidized LDL (μg/mL)	11.3 ± 1.7	8.2 ± 1.6	0.12
**• Dietary data**			
Energy (kcal)	1,801 ± 695	1,798 ± 662	0.98
Saturated-to-unsaturated fatty acids	0.5 ± 0.2	0.5 ± 0.1	0.66
Total fiber (g)	15.5 ± 5.5	15.6 ± 5.5	0.32
Pro-vitamin A carotenoids (μg)	227.4 ± 2.4	207.5 ± 2.3	0.46
Vitamin E (mg)	5.6 ± 2.1	5.9 ± 3.2	0.63
Vitamin C (mg)	62.7 ± 4.6	64.2 ± 2.8	0.89
Fruits (servings)	1.9 ± 0.6	2.2 ± 0.4	0.11
Vegetables (servings)	1.2 ± 0.2	1.6 ± 0.3	0.64

Participants consumed a mean of 1,800 ± 672 kcal/day (50.4 ± 6.8% carbohydrate, 18.1 ± 4.2% protein and 31.2 ± 5.8% fat). Mean energy intake, percentage macronutrients, vitamins and FV were similar between genders. A high proportion of individuals were below recommended levels for micronutrients (98% for pro-vitamin A carotenoids, 89% for vitamin E and 70% for vitamin C). On average, subjects consumed 3.8 ± 2.8 servings/day of FV. Mean time spent on physical activities was 181 min/week, ranging from zero to 1,260 minutes/week.

When individuals were stratified according to servings of FV (Table 2) the male-to-female ratios were similar among the three groups. They also had similar energy intake but, as expected, the higher the FV intake the higher the intake of total fiber and vitamins (data not shown). Mean adiponectin concentration was significantly greater in the highest category of FV intake than in the lowest category. Significant trends for lower mean values of waist circumference (103.4 ± 13.6 vs. 100.1 ± 12.2 vs. 98.2 ± 12.7 cm, p-trend = 0.02) and higher adiponectin concentrations (10.4 ± 1.8 vs. 11.9 ± 1.9 vs. 13.6 ± 2.1 ng/mL, p-trend = 0.02) were detected. No significant difference was found among groups stratified according to tertiles of vitamin intake.

**Table 2:** Clinical and laboratory characteristics of participants, according to groups of fruit and vegetable consumption (n = 205)

	**Servings of fruits & vegetables**			
	**≤ 2.49**	**2.5 to 5**	**≥ 5**	**p for trend**
	**N = 78**	**N = 70**	**N = 55**	
Total intake (kcal)	1859 ± 713	1761 ± 610	1755 ± 702	0.59
Body mass index (kg/m^2^)	31.6 ± 6.0	30.2 ± 5.6	29.9 ± 5.9	0.19
Waist circumference (cm)	103.4 ± 13.6	100.1 ± 12.2	98.2 ± 12.7^**†**^	0.02
Fat mass (%)	33.9 ± 0.6	32.5 ± 0.8	33.9 ± 0.6	0.65
Fasting plasma glucose (mg/dL)	98.4 ± 10.9	100.2 ± 12.7	99.7 ± 10.7	0.62
HOMA-IR^#^	2.1 ± 1.9	1.9 ± 1.8	1.9 ± 1.9	0.36
Triglycerides (mg/dL)	157.0 ± 76.1	146.8 ± 61.1	142.1 ± 60.9	0.42
Non-HDL cholesterol (mg/dL)	160.1 ± 42.7	155.7 ± 40.4	150.9 ± 41.8	0.43
C-reactive protein^#^ (mg/mL)	0.4 ± 0.1	0.3 ± 0.1	0.3 ± 0.1	0.41
Tumor necrosis factor-α^#^ (pg/mL)	10.9 ± 0.5	11.0 ± 0.7	10.6 ± 1.2	0.86
Adiponectin^#^ (ng/mL)	10.4 ± 1.8	11.9 ± 1.9	13.6 ± 2.0^**†**^	0.02
Superoxide dismutase^#^ (U/mL)	41.5 ± 1.3	42.6 ± 1.3	46.1 ± 1.6	0.26
Oxidized LDL^#^ (μg/mL)	6.8 ± 0.6	7.5 ± 0.9	9.9 ± 3.9	0.08

Continuous variables are presented as mean values ± standard deviations.

^†^ p < 0.05 *versus* “group ≤ 2.49”, using post hoc Bonferroni test to correct for multiple comparisons.

^#^ Transformed for analysis.

The oxLDL concentration was correlated to HOMA-IR (r = 0.436, p = 0.03), while SOD concentration was correlated to both adiponectin (r = 0.184, p < 0.01) and CRP (r = −0.198, p < 0.01) concentrations.

Table 3 depicts linear regression models for markers of inflammation, insulin resistance and oxidative stress. The association of adiponectin concentration with pro-vitamin A carotenoids intake observed in the crude model disappeared after adjustments. No association was found between CRP and TNF-α concentrations (data not shown), nor between HOMA-IR and dietary variables. However, direct associations were found between SOD concentrations and FV after adjustments for age, gender, BMI, saturated fat intake, smoking and physical activity (β = 0.172 [0.110-0.688]). Inverse associations of oxLDL concentrations with vitamin C (β = −0.333 [−2.568 – −0.218]) and vitamin E (β = −0.354 [−1.131 – −0.110]) intakes were found in the same models. Similar results were found between oxLDL and intake of FV, but significance disappeared after adjusting for saturated fatty acids, smoking and physical activity.

**Table 3:** **Crude and adjusted linear regression coefficients (95**% **confidence interval) for the associations of dietary variables with markers of inflammation, oxidative stress and insulin resistance**

	**CRP**	**Adiponectin**	**HOMA-IR**	**SOD**	**oxLDL**
Fruits & vegetables	- 0.050	0.106	- 0.021	0.202	- 0.373
	(-0.535 – 0.304)	(-0.138 – 1.287)	(-4.848 – 5.991)	(0.120 – 0.734)	(-1.526– -0.180)
Model 1	- 0.070	0.018	0.007	0.187	-0.471
	(-0.558 – 0.319)	(-0.522 – 0,959)	(-3.609 – 7.749)	(0.100 – 0.715)	(-2.966 – -0.110)
Model 2	-0.071	- 0.010	0.032	0.172	-0.330
	(-0.552 – 0.288)	(-0.624 – 0.775)	(-3.119 – 7.586)	(0.110 – 0.688)	(-3,608 – 0.007)
Pro-vitamin A carotenoids	0.033	0.174	0.017	0.093	0.155
	(-0.283 – 0.456)	(0.041 – 0.349)	(-0.140 – 0.180)	(-0.109 – 0.490)	(-0.410 – 0,186)
Model 1	- 0.023	0.060	0.011	0.055	0.092
	(-0.445 – 0.321)	(-0.091 – 0.235)	(-0.155 – 0.182)	(-0.187 – 0.399)	(-0.390 – 0.227)
Model 2	- 0.027	0.077	0.009	0.084	-0.067
	(-0.442 – 0.296)	(-0.071 – 0.245)	(-0.154 – 0175)	(-0.133 – 0.435)	(-0.275 – 0.312)
Vitamin E	-0.004	0.085	-0.036	0.086	-0.367
	(-0.181 – 0.172)	(-0.028 – 0.120)	(-0.096 – 0.057)	(-0.059 – 0.228)	(-0.051 – 1.190)
Model 1	-0.055	0.009	-0.049	0.079	-0.443
	(-0.261 – 0.120)	(-0.076 – 0.087)	(-0.115 – 0.052)	(-0.067 – 0.225)	(-1,243 – -0.191)
Model 2	-0.063	-0.025	-0.014	0.088	-0.354
	(-0.261 – 0.101)	(-0.092 – 0.064)	(-0.089 – 0.073)	(-0.060 – 0.220)	(-1.131– -0.110)
Vitamin C	-0.011	0.097	-0.002	0.159	-0.319
	(-0.644 – 0.553)	(-0.076 – 0.428)	(-0.262 – 0.257)	(0.049 – 1.013)	(-2.334 – 0.986)
Model 1	-0.043	-0.013	0.017	0.141	-0.433
	(-0.815 – 0.446)	(-0.296 – 0.242)	(-0.242 – 0.312)	(-0.024 – 0.937)	(-2.833 – -0.273)
Model 2	-0.043	-0.030	0.031	0.137	-0.333
	(-0.804 – 0.432)	(-0.325 – 0.206)	(-0.211 – 0.339)	(-0.049 – 0.900)	(-2.568 – -0.218)

Continuous variables used for analysis.

CRP, C-reactive protein; SOD, superoxide dismutase; oxLDL, oxidized LDL.

Model 1 - adjusted for age, gender and BMI.

Model 2 – adjusted for age, gender, BMI, saturated fatty acids intake (per 1000 kcal), smoking and physical activity.

## Discussion

In an at-risk sample of the Brazilian population, this study reinforced a beneficial role of FV, as well as antioxidant vitamins, in the pathophysiological process of cardiometabolic diseases, major causes of mortality worldwide. This assertion is based on findings of inverse associations of oxidative stress and inflammation biomarkers with the intake of these nutrients. It is noteworthy that our results remained significant even after adjusting for variables which reflect a healthy lifestyle, such as physical activity, saturated fat intake and smoking.

We detected a positive association of FV consumption with SOD and an inverse association with oxLDL, although the latter had disappeared in the adjusted model. SOD is an essential enzyme that eliminate superoxide radical (O2–) protecting cells from damage induced by free radicals; on the other hand, it is known that oxidation of the LDL particle increases its atherogenic potential [29]. Our findings are in agreement with a recent cross-sectional study, as well as the Women’s Health Across the Nation in which participants with higher intake of vegetables had lower urinary F2-isoprostanes levels, another biomarker of oxidation [30,31]. Also in intervention studies, individuals who consumed a diet rich in FV had significantly lower concentrations of oxidative stress biomarkers [32,33].

Beneficial properties of FV include an interaction of nutrient and non-nutritive components in whole foods [1-7]. Despite knowing that the combination of nutrients in foods have greater health benefits when combined than when consumed alone [2], each nutrient *per se* might have its own effect on the risk of cardiometabolic diseases. Therefore, our study also focused on the associations of pro-vitamin A carotenoids and vitamins C and E intakes with the biomarkers of oxidative stress. Actually, associations of these vitamins with oxidative stress were previously demonstrated [29,34,35]. In participants of present study, intakes of vitamins C and E were inversely associated with oxLDL concentrations. Lower levels of this atherogenic particle would be particularly desirable taking into consideration the prediabetic condition of the participants, which increases cardiovascular risk [1,2,15,17]. Oxidative stress triggers inflammation stimulating the NF-кB pathway [36-38] and both processes were shown to be strongly associated with insulin resistance [15,17]. Along the same lines, we found associations of oxLDL concentrations with HOMA-IR and of SOD with adiponectin and CRP, supporting a role for these parameters in assessing oxidative and inflammatory states. These findings are in agreement with the results of the Women’s Health Study [39], indicating that pro-inflammatory pathways contribute to the onset and/or aggravation of insulin resistance, a major pathophysiologic basis of cardiometabolic diseases.

Considering that low intake of FV is a major modifiable risk factor contributing the risk for several diseases in epidemiological studies [39-41], for clinical purposes, the number of servings of this food group may be a useful indicator of oxidative status and low grade inflammation. International recommendations have established a minimum of 5 servings of FV per day (equivalent to 400 grams) as part of a healthy diet [1,3]. This was the *rationale* for the categorization of FV intake in the present study. In our sample, number of servings (as well as pro-vitamin A carotenoids and vitamins E and C intakes) fell below this recommendation, since the participants consumed a mean of 3.8 servings/day of FV. This finding was somehow expected since previous studies consistently reported that people living in both developed and developing countries consume low amounts of this food group [3,42]. A minimal intake of 5 servings per day of FV should guarantee the amount necessary to reduce oxidative stress and inflammation, involved in the genesis of cardiometabolic diseases. In fact, in our sample, those individuals who consumed the recommended amount of FV had significantly lower waist circumference and higher adiponectin concentrations. Concordantly, other investigators found that diets with high antioxidant capacity were related to increased adiponectin concentrations [43]. Considering that abdominal adiposity is a central feature for the development of insulin resistance, coupled with the favorable effects of adiponectin on these processes [18-20], this dietary habit should be highly desirable for reducing the cardiometabolic risk of these individuals. HOMA-IR was not able to identify differences in insulin sensitivity across categories of FV intake. Based on our body of findings, we speculate that habit of FV consumption may be not only indicative of a healthy lifestyle which protects against body fat accumulation. FV contents may favor *per se* antioxidant and anti-inflammatory mechanisms, resulting in attenuation of insulin resistance and atherogenesis.

The present study has limitations and strengths. Circulating levels of vitamins were not available whereas the cross-sectional design precluded determination of causality. Since our sample derived from a longitudinal study, sample size was not calculated to test the current associations. Despite a relatively small sample, this proved sufficient to allow detection of important associations of FV intake with oxidative stress, inflammation status and insulin resistance in a population known to be genetically heterogeneous. The application of epidemiologic methods to nutrition is fraught with complications because of the highly interrelated nature of dietary exposures. For this reason, it is difficult to separate out the specific effects of nutrients or foods despite the common practice of examining the role of single nutrients or foods in relation to disease risk. The combination of these nutrients in foods has greater health benefits than the individual nutrients alone. Another limitation of the present study is related to the intrinsic weakness of the available techniques to quantify food intake, since they are dependent of individual report [44]. This context may result in the detection of associations of small magnitudes between nutrients and diseases. Precautions were taken to improve the accuracy of the dietary data collected. Strength of our study was the detailed assessment of dietary intake in this at-risk sample. Individuals provided more reliable diet record data by giving a three-day food record, including a weekend day. It is also noteworthy that data on two markers of antioxidant-oxidative status were available, namely SOD and oxLDL concentrations, given that no single gold-standard is defined in literature.

In summary, the assessment of FV or selected vitamins intakes in clinical settings may be useful to identify oxidative stress and inflammation involved in the genesis of cardiometabolic diseases and for motivating at-risk patients for changing dietary habits. This study supports that international guidelines of ≥ 5 servings per day of FV should be emphasized in an attempt to attenuate underlying mechanisms of chronic metabolic and cardiovascular diseases.

## Abbreviations

BMI: Body mass index; CPR: C-reactive protein; FSP-USP: School of public health of the University of São Paulo; FV: Fruits & vegetables; HOMA-IR: Homeostasis model assessment; oxLDL: Oxidized LDL; SOD: Superoxide dismutase; TNF-α: Tumor necrosis factor alpha.

## Competing interests

The authors declare that they have no competing interests.

## Authors’ contributions

LDF made substantial contributions to conception and design of the study, and participated in acquisition, analysis and interpretation of data and drafted the manuscript. MMP helped in the statistical analysis and writing the manuscript. CRB participated in acquisition of data. LAM participated in interpretation of data and discussion. SRGF participated in the conception, design and coordinated the study; she also helped to draft the manuscript and gave the final approval of the version to be published. All authors read and approved the final manuscript.
